# Impact of Early Nutrition, Physical Activity and Sleep on the Fetal Programming of Disease in the Pregnancy: A Narrative Review

**DOI:** 10.3390/nu12123900

**Published:** 2020-12-20

**Authors:** Jorge Moreno-Fernandez, Julio J. Ochoa, Magdalena Lopez-Frias, Javier Diaz-Castro

**Affiliations:** 1 Department of Physiology, Faculty of Pharmacy, Campus Universitario de Cartuja, E-18071 Granada, Spain; jorgemf@ugr.es (J.M.-F.); maglopez@ugr.es (M.L.-F.); javierdc@ugr.es (J.D.-C.); 2 Institute of Nutrition and Food Technology “José Mataix Verdú”, University of Granada, E-18071 Granada, Spain

**Keywords:** nutritional programming, eclampsia, birth weight, cardiovascular disease, metabolic programming, physical activity, sleep, obesity, pregnancy

## Abstract

Early programming is the adaptation process by which nutrition and environmental factors alter development pathways during prenatal growth, inducing changes in postnatal metabolism and diseases. The aim of this narrative review, is evaluating the current knowledge in the scientific literature on the effects of nutrition, environmental factors, physical activity and sleep on development pathways. If in utero adaptations were incorrect, this would cause a mismatch between prenatal programming and adulthood. Adequate caloric intake, protein, mineral, vitamin, and long-chain fatty acids, have been noted for their relevance in the offspring brain functions and behavior. Fetus undernutrition/malnutrition causes a delay in growth and have detrimental effects on the development and subsequent functioning of the organs. Pregnancy is a particularly vulnerable period for the development of food preferences and for modifications in the emotional response. Maternal obesity increases the risk of developing perinatal complications and delivery by cesarean section and has long-term implications in the development of metabolic diseases. Physical exercise during pregnancy contributes to overall improved health post-partum. It is also interesting to highlight the relevance of sleep problems during pregnancy, which influence adequate growth and fetal development. Taking into account these considerations, we conclude that nutrition and metabolic factors during early life play a key role of health promotion and public health nutrition programs worldwide to improve the health of the offspring and the health costs of hospitalization.

## 1. Introduction

Early programming is defined as the adaptation process by which nutrition and other environmental factors alter in utero fetal development, thereby inducing changes in metabolism and the susceptibility to chronic disease in adulthood [1]. Animal studies showed that the fetus can adapt to malnutrition, altering its hormonal production or the sensitivity of tissues to hormones and insulin has a key role regulating fetal growth, and therefore the need for nutrients [2]. The fetus can alter its metabolism, for example, by changing the oxidation of glucose to that of amino acids, can also redistribute blood flow to major organs like the brain and can even adapt to slower growth to decrease substrate demands. However, contrary to the physiological adaptations that occur in the adult, those of the fetus lead to permanent changes in body structure and function [3].

Early programming begins before conception, because during production of both male and female primordial germ cells, most epigenetic imprints (such as DNA methylation, histone modification, and microRNAs) included in the parent’s DNA are eliminated, although in some cases these imprints remain [4,5]. On the other hand, the development period from preconception to early childhood is the time when epigenetic imprinting of DNA occurs most actively, which leads to alterations in gene expression with lasting effects [6]. Nutrition is, from all the environmental factors, one of the most important quantitatively in the environment-gene relationship, therefore prenatal and postnatal nutrition are the most influential environmental factors during fetal and infant development [7]. In addition, the fetal period has enormous plasticity and capacity to respond to the lifestyle and the maternal environment [8], and nutrition plays a fundamental role in this stage since it induces permanent methylation of DNA [9].

The dietary components (vitamins, micronutrients, amino acids, fats or carbohydrates) can affect the function and expression of genes in the uterus and during the early stages of life by modulating epigenetic mechanisms mediated by the metabolism of folates in the metabolism of carbohydrates or in the transmethylation processes that affect the methylation of DNA, histones or non-coding miRNAs [8]. This is an area of epigenetic research, in which it is studied whether epigenetic alterations induced by the maternal diet program the offspring to be more susceptible to certain pathologies such as cardiovascular diseases, obesity, diabetes [5].

**Figure 1:**
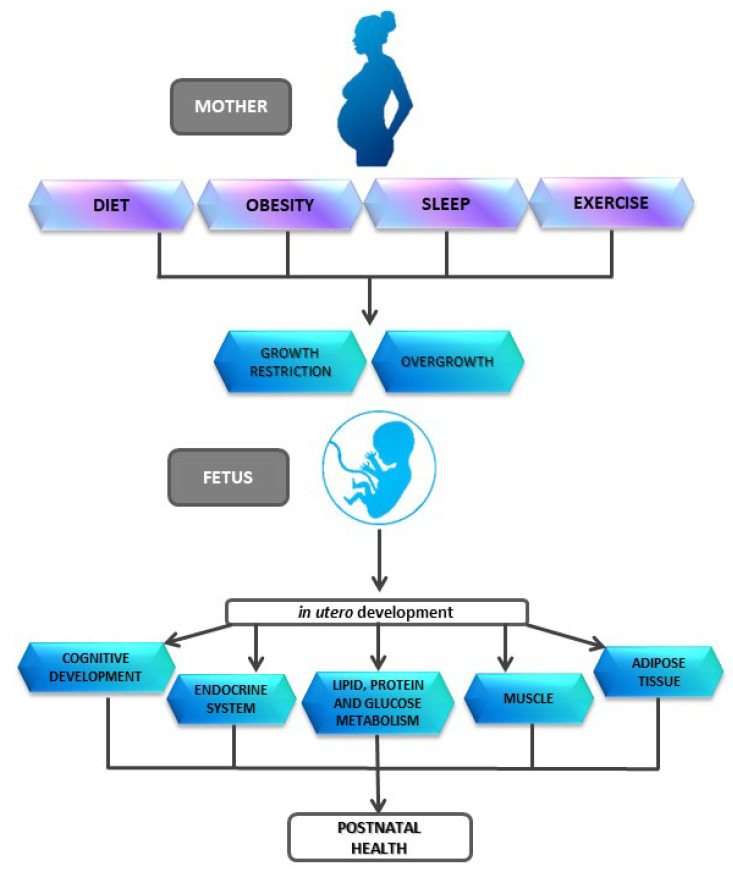
Influence of several factors on offspring development.

The link between the intrauterine environment and the risk of disease in adulthood was first studied in the 1980s, showing that there was a higher mortality from cardiovascular diseases in newborns with low birth weight, data confirmed later by studies that also found an association between low birth weight with an increased risk of obesity, diabetes mellitus type II and hypertension [10,11,12]. Based on these findings, Barker proposed the “thrifty phenotype” hypothesis, also known as the “Barker hypothesis” [13], reporting that exposure to inadequate nutrition during pregnancy and childhood predisposes offspring to developing diabetes mellitus type II. This theory proposes that malnutrition, regardless of the cause, limits the fetus growth by prioritizing blood flow to the brain, thus decreasing the flow to muscle, liver, pancreas and kidneys. In addition, secretion and sensitivity to hormones that promote fetal growth such as insulin or insulin-like factors are reduced [14]. The further development of this hypothesis gave rise to the theory of the origin of health and disease (The Developmental Origins of Health and Disease, DOHaD) [5,12,14], which established that the fetus has capacity to make adaptations based on signals from the in utero environment, thus allowing to improve the immediate survival under adverse postnatal environment. If in utero adaptations are incorrect or significant changes occur in the environment after birth, this would cause a mismatch between prenatal programming and postnatal life and, therefore, induce an increased risk of disease in adulthood [15] (Figure 1).

The first studies that clearly showed the relationship between nutrition during pregnancy and the risk of disease in adulthood were those developed during the 1944 Dutch famine [11]. In these studies, it was observed that the children of women exposed to famine periconceptionally or during the first trimester of gestation presented newborns with similar weights to the children of mothers not exposed to it, however, in adulthood they featured increased risk of cardiovascular disease and diabetes. In those cases, in which exposure to famine took place in more advanced stages of gestation, the birth weight was lower among the children of women exposed to famine, also presenting a higher incidence of hypertension and insulin resistance in the adulthood [11].

There is increasing evidence in the scientific literature showing that embryonic or fetal exposure to nutrients can affect their epigenetics and many of these changes are lasting throughout lifespan [16], and can be the most plausible etiology of pathologies such as some types of cancer, cardiovascular disease or metabolic diseases [17]. However, not only malnutrition affects the epigenome, overnutrition also involves metabolic programming and an increased risk of cardiovascular disease, obesity, hypertension, atherosclerosis and diabetes mellitus [9].

Nutrition and physical activity during pregnancy are correlated, showing that maternal malnutrition can lead to a sedentary life in the offspring [18]. This aspect is of great importance, since physical activity during pregnancy has been positively related to various healthy aspects both in the mother and in the newborn [19]. Recently, growing evidence in the scientific literature, indicates that physical activity is necessary to prevent possible metabolic disturbances in the offspring and also may have a positive correlation with offspring’s neurodevelopment and language (in children from 18 to 60 months) [20].

On the other hand, physiological changes that occur during pregnancy, are associated with changes in sleep architecture. These changes can cause negative effects on the health of the mother and her offspring both at a psychological level, as well as at a metabolic and epigenetic level [21]. Also, sleep duration and quality have an impact in dietary patterns which has a direct impact on the offspring’s health outcomes [22].

Taking into account all the mentioned above, the aim of this narrative review, is evaluating the current knowledge in the scientific literature on the effects of nutrition, environmental factors, physical activity and sleep on development pathways during prenatal growth, and their effects in lifelong metabolism and diseases.

## 2. Materials and Methods

The search dated from 3 January 2020 to 2 September 2020. Website search engines and electronic databases such as PubMed, Scielo and Cochrane were used, including consistent key words such as early programming, metabolic programming, in utero programming, maternal diet, fetal growth. Dates of all publications were noted if available. The inclusion criteria were: manuscripts with expertise in the field of nutrition. Exclusion criteria were: manuscripts written in a language different from English or Spanish, webinars, blogs or podcasts. Keywords included were: “Nutritional programming”; “Birth weight”; “Metabolic programming”; “Physical activity”; “Sleep”; “Pregnancy”. After screening of titles and abstracts, 49 manuscripts were selected for further examination. Subsequently 2 manuscripts were excluded due to lack of relevance, therefore 47 papers were included in this narrative review. Text from each manuscript was extracted into an excel document and reviewed to find repeated issues by the authors. The topics, main objectives and key words were agreed by all authors.

## 3. Maternal Diet and the Influence in Offspring Health

### 3.1. Influence of Maternal Diet on Cognitive Development and Behavior

There are several critical periods for brain development which include the third trimester of gestation to two years of the child’s life. These periods are characterized by great maturation and brain growth [23]. There are other critical periods that also been highlighted as early organizational processes which occur during the first trimester of gestation, they are highly relevant and include cell migration, differentiation, neurogenesis, synaptogenesis, and maturation of neurotransmission pathways [24]. Adequate caloric intake, protein (which avoids embryonic losses, intra-uterine growth restriction, and reduced postnatal growth), and some minerals such as, iron (critical for rapid development and proliferation playing a key role in brain development), zinc (which affects fetal growth), selenium (which avoids risk of infant infection and improves psychomotor score), copper (necessary for rapid growth and cell differentiation for both the mother and fetus) and iodine (which favors thyroid and neurological development), have been noted for their special relevance in these processes. In this sense, some vitamins and other nutrients, such as folate (which helps form the neural tube and cell proliferation), vitamin A (which aids in both cell development and brain growth), choline (which influences stem cell proliferation and brain and spinal cord structure and function) and long-chain fatty acids (which play important roles as precursors of prostaglandins and as structural elements of cell membranes), also play an essential role in these processes [23]. Hence, an inadequate diet, which does not provide these main nutrients in the right amount during this critical period, can alter irreversibly the offspring brain functions and behavior. In this sense, Coo et al. [25] reported modest associations between maternal obesity and offspring performance intelligence quotient, with a weak biologic effect of maternal adiposity in pregnancy on offspring performance intelligence quotient.

With regard to the intake behavior, some studies have shown how exposure to a high-fat diet or cafeteria-style diet at critical moments in development can cause hyperphagia [26,27,28]. The increase in appetite in the offspring has also been evidenced after exposure to caloric restriction in animal models [29,30,31]. In the lifelong, regarding the preference for meals, different investigations point to the existence of critical periods for their programming. Thus, studies conducted in humans exposed to famines in the perinatal period or with restricted intrauterine growth have shown, in later stages, a high preference for food rich in fats [32] or rich in sugars [33]. In relation to critical periods, some research considers pregnancy to be a particularly vulnerable period for the development of food preferences, as a high correlation was found between maternal energy and macronutrient intake in pregnancy, with the intake of children at 10 years old [34]. Other studies also point to the early postnatal period, showing a positive relationship between the type of food to which children have been exposed during pregnancy and lactation, with their preferences in the weaning period [35], and also showing how exposure to certain flavors during lactation increases the preference for specific foods in the childhood such as broccoli in those fed hydrolysate or soy formulas, meanwhile this food was not preferred by those who were fed milk formulas [36].

Apart from the alterations in the intake behavior, modifications in the emotional response have been documented in animal models after exposure to a hypercaloric maternal diet [37,38,39], a protein restriction diet [40,41] or a hypocaloric diet [42,43]. In general, it has been shown that perinatal exposure to a high fat diet increases anxiety-related behaviors, this effect being similar regardless of whether the animals have been exposed during fetal or early postnatal life [37,39,44]. In contrast, in some studies carried out in animal models of caloric restriction, exposure during early pregnancy and/or during lactation has been shown to increase anxiety-related behaviors compared to other stages of pregnancy [42,43]. In the case of protein restriction, it has been found, in general, that animals exposed to it during gestation exhibit a greater number of exploratory behaviors or great impulsivity [45], but the opposite effect has also been found when protein malnutrition includes both lactation and gestation [41]. Furthermore, in these models, modifications in behaviors related to depression have been described, including problems related to learning, memory, neuromuscular coordination, and behavioral development, although the underlying mechanisms affected are unknown [41].

As regards human studies, in children with low birth weight, a high risk in suffering from anxiety disorders and/or mood disorders has also been reported [46]. Furthermore, a high correlation has been found between the preconception body mass index and maternal obesity with inattention symptoms and negative emotionality in children [47], establishing a link between obesity and emotional difficulties, as fetal brain development depends on maternal substrates supply. Studies of changes in maternal diet and epigenetic gene regulation have focused primarily on the hypothalamus, relating the increased risks of obesity faced by offspring of poor-quality maternal diets. There are a high number of alterations in hypothalamic subregions by gestational diets, including epigenetic mechanisms. Excessive feeding in rodents, severely reduced litter size, led to methylation of the proopiomelanocortin (POMC) gene in the hypothalamus, and induced dysfunctions in homeostatic feeding mechanisms and energy balance [48]. In human environments there is an equally important role for hedonic and motivational feeding, which is supported by brain regions that are important for reward function. In this case, the underlying mechanisms can be explained because overweight in pregnancy increases concentrations of insulin, interleukin-6, and leptin and placental transfer of glucose which increases the risk for neurobehavioral impairments, inducing disturbed maternal metabolic function, which could also support the hypothesis that maternal metabolic status increases the risk of develop behavioral disturbances in children [47,49].

### 3.2. Role of Malnutrition in Development and Lifelong Consequences

Intrauterine growth retardation occurs more frequently in the context of placental insufficiency [50], generally related to preeclampsia or hypertension [51], although it can also occur as a result of unhealthy maternal diet [51,52]. Fetus undernutrition or malnutrition causes intrauterine growth retardation, inducing low birth weight. Regardless of the causative events, poor intrauterine nutrients supply activates a process in which the fetus responds through physiological adaptations, which involve optimizing the bioavailability of nutrients and diverting blood supply to the brain and other key organs. These adaptations increase the survival rate, but result in a delay in growth and have detrimental effects on the development and subsequent functioning of the organs, which affect the renal, cardiovascular, respiratory, and endocrine systems [51,53,54]. Bacchetta et al. [53] examined the impact of intrauterine growth retardation on renal function in preterm infants, finding that infants with intrauterine growth retardation had the least developed renal system, with fewer nephrons than infants with normal intrauterine growth. These authors also found that children affected by intrauterine growth retardation had higher glomerular filtration rates, which means that the nephrons were working more forcibly. These two factors predispose children with intrauterine growth retardation to future kidney failure. Similar effects affect the development and function of other organs. For example, several authors have studied the affectation in the development of pancreatic beta cells in response to fetal programming during intrauterine growth retardation [50]. This situation leads to a reduction in the insulin secretion and a predisposition to glucose intolerance in the adulthood [50]. Fetal malnutrition also predisposes to cardiac pathologies [54] and lung disease [51]. Therefore, the effects of fetal programming help the fetus survive in adverse conditions, leading to mechanisms of nutritional saving and the ability to survive with restricted nutrition, although with adverse consequences latter in life [13]. When nutrition is no longer restricted, in the postnatal period, the homeostasis of energy balance is profoundly altered predisposes the offspring to suffer insulin resistance, obesity and chronic diseases such as diabetes and hypertension [13].

In relation to micronutrients, there is consensus on the scientific literature about the benefit of iodine, iron and folic acid supplements before and during pregnancy. This supplementation must be provided by a healthcare professional, since self-medication with these preparations can cause health problems in the pregnant woman and the fetus [55,56,57]. Vitamin B_12_ and especially folic acid deficiency are the most important causes of nutritional anemia. The manifestations of its deficiencies are due to the decrease in nucleic acid synthesis, altering nuclear maturation and preferentially affecting cells with rapid proliferation. The most common signs and symptoms are megaloblastic anemia, risk of premature delivery, low birth weight, neural tube closure defects, and spina bifida [58,59].

Iron is necessary to prevent anemia which is associated with an increased risk of negative consequences during pregnancy: low birth weight and premature delivery [60]. It is also important for the development of the fetus and the placenta. Iron preparations should be ingested under fasting conditions and their absorption is improved in presence of vitamin C. Iodine is essential for the synthesis of thyroid hormone, essential for the development of all organs, especially the central nervous system, so that a Iodine deficiency has detrimental effects on the fetus. WHO advises a daily intake of 250 μg for pregnant women and during lactation (Leung et al., 2011). Micronutrient supplementation before and during pregnancy is associated with an increase in birth weight, a decrease in risk of preterm birth, gestational hypertension and preeclampsia and even improvement of the mental development of the offspring [61].

### 3.3. Metabolic Syndrome and Nutritional Programming

The relationship between episodes of early nutritional restriction and the subsequent appearance of pathologies associated with the metabolic syndrome, such as obesity and type II diabetes, has been revealed through different epidemiological studies in humans [62,63,64] and experimental studies carried out on laboratory animals [65,66]. It has been suggested that the gene pool acquired during evolution would have sensitized certain individuals to develop more markedly, different metabolic pathologies associated with overfeeding and sedentary lifestyle habits, is what is known as the hypothesis of the “thrifty genotype” [67]. Subsequently, and in a complementary way, the thrifty phenotype hypothesis was reported by Hales and Barker [13,68], and according to this, a deficient nutritional contribution during development would propitiate the programming of certain genes and metabolic processes, with the aim of accommodating the organism to a limited supply of nutrients, conferring a survival advantage [69]. However, these adaptations would increase vulnerability to subsequent obesogenic or diabetogenic stimuli [70,71]. This hypothesis was formulated after finding, through numerous epidemiological studies [62,72,73], that different communities subjected to nutritional restriction during critical stages of development were more likely to suffer pathologies associated with metabolic syndrome during adulthood.

The fact that not all subjects develop accelerated growth after an episode of early nutritional restriction, and that it differs in terms of the severity of its consequences in adulthood, raises the possibility that both hypotheses, thrifty genotype and phenotype, share essential aspects when explaining the metabolic programming process. In any case, it seems evident, from the numerous studies carried out to date, that the nutritional environment during the perinatal period represents a key stage in the development of pathologies in adults. Also, in recent years, a branch of computational biology, epigenetics, tries to shed light on the causes that would cause the activation or deactivation of certain genes involved in the subsequent development of these pathologies [74].

The hypothesis that nutritional restriction during perinatal growth is associated with the appearance of metabolic diseases in adulthood has been investigated in different animal models [65,75,76]. In these models, intrauterine growth restriction leads to abnormalities in the endocrine function of multiple glands and hormonal systems of term fetuses, which continue into adulthood. A vast majority of these studies suggest that during chronic nutritional restriction the mechanisms that regulate the metabolism of nutrients develop a marked tendency towards their accumulation, prioritizing anabolic processes over catabolic ones, that is, tissues sensitive to action of insulin show different adaptations that increase the action of it. Among others, there has been an increase in the translocation of GLUT-4 (glucose transporter type 4) in skeletal muscle [77,78] and an increase in glucose uptake and lipogenesis in adipose tissue [79,80,81]. However, animals subjected to nutritional restriction show a decrease in circulating insulin levels, possibly with the aim of compensating for the increased sensitivity to the hormone and thus allowing the correct supply of glucose to the brain.

Despite the fact that in recent years there have been important advances in this field, the intrinsic causes that motivate the appearance of diseases in adults, in relation to their previous nutritional status, have not yet been fully clarified. However, in a scenario of early nutritional restriction and subsequent feedback with a fat diet, the accumulation of nutrients in the form of fat appears to be predominant over its use for energy, which is known as accelerated growth of adipose tissue or catch-up fat [82,83]. This energy saving mechanism aims to ensure a nutritional reserve for subsequent periods of food shortage. Therefore, in developing societies, where less balanced and more abundant and energetic diets are consumed, this saving feature seems to be decisive in the exponential growth of obesity and type 2 diabetes [84,85].

### 3.4. Excessive Calories Consumption in the Maternal Diet and Influence on the Development

The focus on programming research has evolved from studies of malnutrition to those of excessive consumption of maternal calories and obesity, a major global public health problem that affects more than 50% of women in many countries. Maternal obesity (either before pregnancy or during pregnancy), could induce a critical situation known as “excessive fetal nutrition” [14], that increases the risk of obesity and complications in the offspring. There are plenty of studies in animals and observational studies in humans, which support this hypothesis [14,86,87,88]. Consistent associations have been identified [87] between increased adiposity in the offspring with maternal body mass index before pregnancy, increased gestational weight, gestational diabetes, and excessive birth weight. The hypothesis of excessive fetal nutrition is supported by the neonates of mothers who lose weight after bariatric surgery have a lower risk of obesity compared to babies of the same mother born before surgery [89].

Prevention strategies are of vital importance to avoid the persistence of obesity during growth and maturation and in adulthood, especially for the prevention of comorbidities associated with the medium and long term [90,91]. Evidence shows the effects of programming antenatal care on subsequent obesity and the appearance of chronic diseases related with nutrition [92]. Weight gain in pregnant women that influences in the fetus metabolism, as well as low and high birth weight, followed by early and excessive weight gain during the first two years of life, are associated with a significant increase in risk of subsequent obesity [93,94]. Even though an overweight or obese child or adolescent will not always be an obese adult, this condition is associated with high risk of persistence in adulthood: obesity a the two-year-old increases the risk 1.3 times, while if maintained until 15–17 years, the risk is 17 times higher, compared to their eutrophic pairs [95].

Maternal nutritional status before and during pregnancy has a key role on fetal growth, increasing the possibility of developing obesity in later life, due to the programming effect during this stage, as demonstrated by epidemiological and experimental studies [96]. Changes in fetal gene expression, which are induced by maternal nutrition, are called epigenetic nutritional changes and appear to be associated with DNA methylation. Prolonged exposures to diets that influence chromatin remodeling and DNA methylation can induce permanent epigenetic changes in the genome. Decreased methylation may be related to slow intrauterine growth. Maternal nutrient restrictions [97] and excessive caloric intake [98] are related to the nutritional epigenetic programming of metabolic disorders [97].

It has been shown that maternal dietary intake and phenotypic adaptations in the offspring have a U-shaped relationship, as demonstrated by observational studies in humans, indicating that alterations that affect normal growth patterns, increasing the risk of obesity, type II diabetes mellitus and metabolic disorders in the offspring, due to both deprivation or nutrients excess in utero [99]. In this sense, higher degrees of maternal obesity increase the risk of developing perinatal complications, such as preeclampsia, macrosomia, and delivery by cesarean section [100]. As is known, obese women have a higher predisposition to lipotoxicity, metabolic dysregulation, oxidative stress and inflammation that those with normal body mass index, which can be exacerbated by fat accumulation during pregnancy [101,102].

As previously mentioned, excessive weight of the pregnant mother affects her health and that of the offspring. Overweight and maternal obesity are highly correlated with neonatal fat mass in the first hours of life [103], and overweight in the adult stage [104]. On the other hand, obese women have between 3–6 times the risk of having macrosomic newborns, being a determining factor for overweight and obesity during childhood and adolescence [105]. On the other hand, adequate birth weight has been associated with a higher lean mass and lower fat mass compared to low birth weight infants [106]. High prenatal intake of energy, protein and micronutrient deficiency has been linked to an increased risk of obesity in the adult stage of the offspring and obese women consume highly energetic diets but poor in essential micronutrients [107].

Taking into account the evidence in the scientific literature that shows the impact of maternal nutrition on the risk of chronic diseases in the adult life of the offspring, several researchers have undertaken studies to determine possible strategies that can reverse these effects from early stages of life. The objective of these studies is therefore to prevent obesity and related diseases in the future adult life of individuals characterized by poor metabolic programming. In order to reverse the effects of programming, these strategies must be carried out in critical stages of fetal and/or neonatal life, that is, in periods that correspond to the development and/or maturation of different systems and are thus liable to exert the effects of each strategy and reverse the programming.

Due to its relevance as a neurotrophic factor and regulator of energy homeostasis, leptin supplementation in postnatal life is an important reversal strategy, which has many evidences in favor of its effectiveness. During the second week of life, a surge of leptin occurs that coincides with the development of hypothalamic circuits. The origin of this leptin surge is still a matter of controversy, although it is suggested that neonatal leptin comes from breast milk, through lactation, since in that period the adipose tissue is still immature. This fact has been supported by the rapid decrease in leptin levels in neonates separated from the mother at birth, as well as by the increased risk of obesity in children fed a lactating formula instead of breastfeeding [108]. Therefore, a leptin supplementation in this period, may have the ability to reverse some of the neuroanatomical effects and other characteristics of the obese phenotype associated with the absence of the leptin surge. Specifically, intraperitoneal injections of leptin (10 mg/kg) between day 4 and 12 of life in leptin-deficient mice (ob/ob mice), have shown a reversal in the altered development of hypothalamic circuits related to hunger [109]. Similarly, daily injections of subcutaneous leptin (2.5 µg/g/day) during the first 13 days of life in offspring of rats subjected to a 30% caloric restriction [110] during pregnancy, have showed a normalization in caloric intake, locomotor activity, body weight, fat mass and in glucose, insulin and leptin levels in adult life.

The metabolic dysfunctions caused by the lack of leptin signaling during early life involve changes that affect energy homeostasis, reproduction and brain development. Although the complete absence of leptin signaling is rarely found in humans, both undernutrition and overnutrition are able to produce significant changes in leptin concentrations, leptin sensitivity or in the development of neurocircuits that regulate energy homeostasis [111,112,113]. Therefore, intrauterine or early postnatal changes in energy balance have been consistently linked with obesity and other metabolic diseases in adulthood [114,115,116].

Konieczna et al. [109] reported the effectiveness of the administration of oral doses of leptin through lactation in terms of reversing poor programming. This treatment was administered to offspring of rats descended from mothers subjected to a 20% caloric restriction during the first 12 days of pregnancy, associated with alterations in the hypothalamic circuits as a consequence of said conditions. Specifically, supplementation consisted of administering 1 ng of leptin on day 1 of life to the offspring and gradually increasing the dose to reach 43.8 ng on day 20 of life. The results of this study showed that this oral administration of leptin during lactation led to the reversal of neuroanatomical disorders, produced as a consequence of maternal caloric restriction.

## 4. Exercise and Metabolic Programming

There is evidence that maternal malnutrition in rats results in decreased physical activity, leading to a sedentary life in the offspring. Specifically, a study carried out under conditions of maternal protein restriction during pregnancy showed a significant decrease in voluntary locomotive activity in their offspring [18,19]. Thus, it has been suggested that moderate exercise activity is necessary to prevent possible metabolic disturbances in the offspring and also may have a positive correlation with offspring’s neurodevelopment and language (in children from 18 to 60 months) [20]. It also has been reported that a combination of aerobic-resistance exercises of moderate-to-vigorous intensity in pregnant women from the 17th week until delivery (3 days/week, 60 min/session) is a complementary-alternative tool to modulate the immune status, avoiding future immunometabolic impairments and prevent pregnancy complications [117].

Physical exercise causes physiological changes that depend on the type, intensity and duration of the effort, as well as the physical training, age, gender and nutritional status of the individual. In pregnant women, it often happens that they decrease their physical activity and restrict physical exercise. Recently, new knowledge about the risk/benefit ratio of exercise during pregnancy has emerged, making consensus and recommendations essential to guide public health policies [118]. Training during pregnancy is safe and positive as reported by several studies [119,120,121]. Exercise improves overall health status in pregnant women and they feel better and experience fewer pregnancy-related symptoms [121], being the cardiovascular responses to exercise in pregnant women similar to those observed in non-pregnant women [120].

Furthermore, it is known that regular physical exercise of moderate intensity, and early onset (first 20 weeks of gestation), increases placental blood perfusion and functional capacity. In this way, exercise can prevent placental abnormalities, one of the underlying causes of preeclampsia leading to decreased cell perfusion and placental hypoxia. In addition, the resulting oxidative stress causes endothelial dysfunction can trigger high blood pressure and preeclampsia, but also induce the development of gestational diabetes [122]. In this sense, as physical exercise renews placental perfusion, improves the supply of nutrients necessary for fetal development, and substantially reduces the risk of developing pre-eclampsia [123].

All of these cardiovascular changes contribute to overall improved health post-partum, decreasing complications during labor and delivery, time for maternal recovery and lifelong adverse conditions [124]. Therefore, there are numerous maternal and pregnancy benefits related exercise while pregnant that can have a clear beneficial impact on adulthood health.

## 5. Sleep and Metabolic Programming

Sleep duration and quality has an impact in dietary patterns. This new area of research in nutritional sciences studying the impact of the timing of eating on health outcomes is called chrono-nutrition, and combines elements from nutritional research with chrono-biology which has a direct impact on health outcomes [22]. Physiological changes that occur during pregnancy are also associated with changes in sleep architecture. Total sleep time increases slightly in the first trimester of pregnancy compared to the non-pregnant woman and increases in a more pronounced way during the third trimester. Sleep disturbances are present during all gestation, but during the third trimester a greater impact on the quality and quantity of sleep is observed [125] in this period, the total sleep time increases approximately half an hour and is accompanied by a reduction in the quality of sleep [126]. Alterations of the circadian rythm can lead to various adverse reproductive outcomes. In addition to the clock, lipid metabolism may also play a key role in fetal development. In the study of Kovac et al. [127], they found a modest interaction between a single nucleotide polymorphism in PER3 and triglycerides on the association with preterm birth. Sleep disturbances during pregnancy might be detrimental to both mother and offspring by promoting increased risk at the behavioural, electrophysiological, metabolic and epigenetic levels and are associated with some hormonal changes, including: increased progesterone levels (causing fatigue and daytime sleepiness), increased estrogen levels (which selectively decrease REM sleep activation in the preoptic ventrolateral area), alterations in cortisol secretion (which decreases REM sleep and increases slow wave sleep), alterations in prolactin levels (which exacerbates slow wave sleep), alterations in growth hormone levels (associated with the initiation and maintenance of slow wave sleep) and these changes play an important role in the growth and development of the fetus [21,128]. Although these changes obey a normal physiological process in pregnant women, they can generate complications, especially when obesity occurs, since the prevalence of sleep apnea is higher. Furthermore, in several studies it has been described that women with pre-eclampsia, a condition associated with maternal obesity, more frequently present respiratory disturbances, nocturnal hypoxia events and obstructive sleep apnea [129,130]. In addition, sleep is one of the primary activities of the brain during early development and plays an important role in healthy cognitive and psychosocial development during the childhood [131].

Serum melatonin levels in pregnant women have a diurnal rhythmicity with significant increases towards the end of pregnancy [132]. Melatonin is transferred from the maternal blood into the fetal circulation with high efficiency, in spite of the fetus can produce its own melatonin with circadian rhythmicity [133,134]. The placental dysfunction, which is suggested as a consequence of the decrease in melatonin levels or of the alterations in its secretion rhythm, raises the possibility that the disruption of light-dark cycles during pregnancy has long-term metabolic consequences, including an increase in adiposity, hyperleptinemia, hyperinsulinemia and glucose intolerance [135,136]. Although the mechanisms through which these alterations are generated have not been fully elucidated, it has been suggested that melatonin may play an important role in the establishment of metabolic alterations in adulthood [136]. Maternal melatonin can affect the functions of the placenta and the fetal organism [137]. Melatonin has been reported to counteract the generation of free radicals by avoiding the oxidative stress that characterizes the early stages of placental abnormalities. In this sense, women with preeclampsia who show low levels of melatonin have deficiencies in the passage of nutrients and oxygen supply to the fetus, which promotes complications of pregnancy, premature delivery and low birth weight [137]. On the other hand, depending on its secretion rhythm, the melatonin produced by the maternal organism is easily transported through the placenta and reaches the fetal circulation. It is essential to regulate some functions during development: maturation of the nervous system, regulation of neonatal temperature, determination of the circadian rhythms of other tissues such as the liver, synchronization of the circadian cycles of the fetus [137], mediation in the expression of the leptin gene in adipocytes through its interaction with specific receptors [138], which promotes the control of food intake and favors the regulation of body weight [139,140] as well as the adequate function of the pancreas and glucose metabolism [141]. It is interesting, therefore, to highlight the relevance of sleep problems during pregnancy, which influence adequate growth and fetal development, early preventing the development of obesity and metabolic diseases. Finally, melatonin supplementation in pregnancy and lactation may serve as a reprogramming strategy against the development of cardiovascular and neurological diseases [142].

## 6. Conclusions

Nutritional, endocrine, metabolic and lifestyle factors in the mother during pregnancy profoundly influence the growth and development of the fetus and progeny, altering tissue function, facts that can affect the susceptibility to several disorders latter in life. Nutrition in early life establishes profound physiological and metabolic changes that determine the risk of diseases that occur with aging. Malnutrition of the fetus causes intrauterine growth retardation, inducing a low birth weight and has detrimental effects on the development and subsequent functioning of the organs, which affect the renal, cardiovascular, respiratory, and endocrine systems. On the other hand, excessive maternal nutrition causes macrosomia, with greater adiposity and a trend towards childhood obesity, type 2 diabetes mellitus and cardiovascular diseases. Intrauterine hyperglycemia is associated with an increased risk of obesity, metabolic disease in offspring. Moderate exercise activity is necessary to prevent possible metabolic disturbances in the offspring and also physical activity practice may have a positive association with language offspring’s neurodevelopment and is a complementary tool to modulate the immune status of pregnant women and their newborns. Finally, sleep problems during pregnancy influence adequate growth and fetal development, early preventing the development of obesity and metabolic diseases. However, this narrative review has some limitations because describes and appraises published articles available, but may include potential selection and evaluation biases. This is in contrast to systemic reviews and meta-analyses, whose queries, criteria, and article selection processes are well-defined. Despite these limitations, our review may provide novel insights into early development factors and also provides strategies for prevention and management of the risk of metabolic disorders in adulthood and potential impacts on the health of the next generation.

## References

[1] Barker D.J., Gluckman P.D., Godfrey K.M., Harding J.E., Owens J.A., Robinson J.S. (1993). Fetal nutrition and cardiovascular disease in adult life. Lancet.

[2] Thompson J.A., Regnault T.R. (2011). In utero origins of adult insulin resistance and vascular dysfunction. Semin. Reprod. Med..

[3] Johnston F.E. (1995). Mothers, babies, and disease in later life. By D. J. P. Barker. London: British Medical Journal Group, 1994. 34.95£ (cloth). Am. J. Hum. Biol..

[4] Palmer D.J., Huang R.C., Craig J.M., Prescott S.L. (2014). Nutritional influences on epigenetic programming: Asthma, allergy, and obesity. Immunol. Allergy Clin. N. Am..

[5] Lee H.S. (2015). Impact of Maternal Diet on the Epigenome during In Utero Life and the Developmental Programming of Diseases in Childhood and Adulthood. Nutrients.

[6] Simeoni U., Yzydorczyk C., Siddeek B., Benahmed M. (2014). Epigenetics and neonatal nutrition. Early Hum. Dev..

[7] Toca Mdel C., Tonietti M., Vecchiarelli C. (2015). [Prenatal and postnatal nutrition: Long term impact on health]. Arch. Argent. Pediatr..

[8] Chango A., Pogribny I.P. (2015). Considering maternal dietary modulators for epigenetic regulation and programming of the fetal epigenome. Nutrients.

[9] Zheng J., Xiao X., Zhang Q., Yu M. (2014). DNA methylation: The pivotal interaction between early-life nutrition and glucose metabolism in later life. Br. J. Nutr..

[10] Tarry-Adkins J.L., Ozanne S.E. (2011). Mechanisms of early life programming: Current knowledge and future directions. Am. J. Clin. Nutr..

[11] Lillycrop K.A., Burdge G.C. (2012). Epigenetic mechanisms linking early nutrition to long term health. Best Pract. Res. Clin. Endocrinol. Metab..

[12] Wang G., Walker S.O., Hong X., Bartell T.R., Wang X. (2013). Epigenetics and early life origins of chronic noncommunicable diseases. J. Adolesc. Health.

[13] Hales C.N., Barker D.J.P. (2001). The thrifty phenotype hypothesis: Type 2 diabetes. Br. Med. Bull..

[14] Fall C.H.D. (2013). Fetal programming and the risk of noncommunicable disease. Indian J. Pediatrics.

[15] Vickers M.H. (2014). Early life nutrition, epigenetics and programming of later life disease. Nutrients.

[16] Lane R.H. (2014). Fetal programming, epigenetics, and adult onset disease. Clin. Perinatol..

[17] Wei Y., Schatten H., Sun Q.Y. (2015). Environmental epigenetic inheritance through gametes and implications for human reproduction. Hum. Reprod. Update.

[18] Bellinger L., Sculley D.V., Langley-Evans S.C. (2006). Exposure to undernutrition in fetal life determines fat distribution, locomotor activity and food intake in ageing rats. Int. J. Obes..

[19] Collings P.J., Farrar D., Gibson J., West J., Barber S.E., Wright J. (2020). Associations of Pregnancy Physical Activity with Maternal Cardiometabolic Health, Neonatal Delivery Outcomes and Body Composition in a Biethnic Cohort of 7305 Mother–Child Pairs: The Born in Bradford Study. Sports Med..

[20] Niño Cruz G.I., Ramirez Varela A., da Silva I.C.M., Hallal P.C., Santos I.S. (2018). Physical activity during pregnancy and offspring neurodevelopment: A systematic review. Paediatr. Perinat. Epidemiol..

[21] Pires G.N., Benedetto L., Cortese R., Gozal D., Gulia K.K., Kumar V.M., Tufik S., Andersen M.L. (2020). Effects of sleep modulation during pregnancy in the mother and offspring: Evidences from preclinical research. J. Sleep Res..

[22] Pot G.K. (2018). Sleep and dietary habits in the urban environment: The role of chrono-nutrition. Proc. Nutr. Soc..

[23] Georgieff M.K. (2007). Nutrition and the developing brain: Nutrient priorities and measurement. Am. J. Clin. Nutr..

[24] Walker C.D. (2005). Nutritional aspects modulating brain development and the responses to stress in early neonatal life. Prog. Neuropsychopharmacol. Biol. Psychiatry.

[25] Coo H., Fabrigar L., Davies G., Fitzpatrick R., Flavin M. (2019). Are observed associations between a high maternal prepregnancy body mass index and offspring IQ likely to be causal?. J. Epidemiol. Community Health.

[26] Nivoit P., Morens C., Van Assche F.A., Jansen E., Poston L., Remacle C., Reusens B. (2009). Established diet-induced obesity in female rats leads to offspring hyperphagia, adiposity and insulin resistance. Diabetologia.

[27] Howie G.J., Sloboda D.M., Kamal T., Vickers M.H. (2009). Maternal nutritional history predicts obesity in adult offspring independent of postnatal diet. J. Physiol..

[28] Ong Z.Y., Muhlhausler B.S. (2011). Maternal “junk-food” feeding of rat dams alters food choices and development of the mesolimbic reward pathway in the offspring. FASEB J. Off. Publ. Fed. Am. Soc. Exp. Biol..

[29] Breton C., Lukaszewski M.A., Risold P.Y., Enache M., Guillemot J., Rivière G., Delahaye F., Lesage J., Dutriez-Casteloot I., Laborie C. (2009). Maternal prenatal undernutrition alters the response of POMC neurons to energy status variation in adult male rat offspring. Am. J. Physiol. Endocrinol. Metab..

[30] Palou M., Priego T., Sánchez J., Palou A., Picó C. (2010). Sexual dimorphism in the lasting effects of moderate caloric restriction during gestation on energy homeostasis in rats is related with fetal programming of insulin and leptin resistance. Nutr. Metab..

[31] Manuel-Apolinar L., Rocha L., Damasio L., Tesoro-Cruz E., Zarate A. (2014). Role of prenatal undernutrition in the expression of serotonin, dopamine and leptin receptors in adult mice: Implications of food intake. Mol. Med. Rep..

[32] Lussana F., Painter R.C., Ocke M.C., Buller H.R., Bossuyt P.M., Roseboom T.J. (2008). Prenatal exposure to the Dutch famine is associated with a preference for fatty foods and a more atherogenic lipid profile. Am. J. Clin. Nutr..

[33] Ayres C., Agranonik M., Portella A.K., Filion F., Johnston C.C., Silveira P.P. (2012). Intrauterine growth restriction and the fetal programming of the hedonic response to sweet taste in newborn infants. Int. J. Pediatrics.

[34] Brion M.-J.A., Ness A.R., Rogers I., Emmett P., Cribb V., Davey Smith G., Lawlor D.A. (2010). Maternal macronutrient and energy intakes in pregnancy and offspring intake at 10 y: Exploring parental comparisons and prenatal effects. Am. J. Clin. Nutr..

[35] Mennella J.A., Griffin C.E., Beauchamp G.K. (2004). Flavor programming during infancy. Pediatrics.

[36] Mennella J.A., Beauchamp G.K. (2002). Flavor experiences during formula feeding are related to preferences during childhood. Early Hum. Dev..

[37] Sullivan E.L., Grayson B., Takahashi D., Robertson N., Maier A., Bethea C.L., Smith M.S., Coleman K., Grove K.L. (2010). Chronic consumption of a high-fat diet during pregnancy causes perturbations in the serotonergic system and increased anxiety-like behavior in nonhuman primate offspring. J. Neurosci..

[38] Peleg-Raibstein D., Luca E., Wolfrum C. (2012). Maternal high-fat diet in mice programs emotional behavior in adulthood. Behav. Brain Res..

[39] Sasaki A., de Vega W.C., St-Cyr S., Pan P., McGowan P.O. (2013). Perinatal high fat diet alters glucocorticoid signaling and anxiety behavior in adulthood. Neuroscience.

[40] Reyes-Castro L.A., Rodriguez J.S., Charco R., Bautista C.J., Larrea F., Nathanielsz P.W., Zambrano E. (2012). Maternal protein restriction in the rat during pregnancy and/or lactation alters cognitive and anxiety behaviors of female offspring. Int. J. Dev. Neurosci..

[41] Belluscio L.M., Berardino B.G., Ferroni N.M., Ceruti J.M., Cánepa E.T. (2014). Early protein malnutrition negatively impacts physical growth and neurological reflexes and evokes anxiety and depressive-like behaviors. Physiol. Behav..

[42] Levay E.A., Paolini A.G., Govic A., Hazi A., Penman J., Kent S. (2008). Anxiety-like behaviour in adult rats perinatally exposed to maternal calorie restriction. Behav. Brain Res..

[43] Akitake Y., Katsuragi S., Hosokawa M., Mishima K., Ikeda T., Miyazato M., Hosoda H. (2015). Moderate maternal food restriction in mice impairs physical growth, behavior, and neurodevelopment of offspring. Nutr. Res..

[44] Bolton J.L., Bilbo S.D. (2014). Developmental programming of brain and behavior by perinatal diet: Focus on inflammatory mechanisms. Dialogues Clin. Neurosci..

[45] Almeida S.S., Tonkiss J., Galler J.R. (1996). Prenatal protein malnutrition affects exploratory behavior of female rats in the elevated plus-maze test. Physiol. Behav..

[46] Hack M., Youngstrom E.A., Cartar L., Schluchter M., Taylor H.G., Flannery D., Klein N., Borawski E. (2004). Behavioral outcomes and evidence of psychopathology among very low birth weight infants at age 20 years. Pediatrics.

[47] Rodriguez A. (2010). Maternal pre-pregnancy obesity and risk for inattention and negative emotionality in children. J. Child. Psychol. Psychiatry.

[48] Plagemann A., Harder T., Brunn M., Harder A., Roepke K., Wittrock-Staar M., Ziska T., Schellong K., Rodekamp E., Melchior K. (2009). Hypothalamic proopiomelanocortin promoter methylation becomes altered by early overfeeding: An epigenetic model of obesity and the metabolic syndrome. J. Physiol..

[49] Grissom N.M., Reyes T.M. (2013). Gestational overgrowth and undergrowth affect neurodevelopment: Similarities and differences from behavior to epigenetics. Int. J. Dev. Neurosci. Off. J. Int. Soc. Dev. Neurosci..

[50] Portha B., Chavey A., Movassat J. (2011). Early-life origins of type 2 diabetes: Fetal programming of the beta-cell mass. Exp. Diabetes Res..

[51] Salam R.A., Das J.K., Bhutta Z.A. (2014). Impact of intrauterine growth restriction on long-term health. Curr. Opin Clin. Nutr. Metab. Care.

[52] Torreggiani M., Fois A., D’Alessandro C., Colucci M., Orozco Guillén A.O., Cupisti A. (2020). Of Mice and Men: The Effect of Maternal Protein Restriction on Offspring’s Kidney Health. Are Studies on Rodents Applicable to Chronic Kidney Disease Patients? A Narrative Review. Nutrients.

[53] Bacchetta J., Harambat J., Dubourg L., Guy B., Liutkus A., Canterino I., Kassaï B., Putet G., Cochat P. (2009). Both extrauterine and intrauterine growth restriction impair renal function in children born very preterm. Kidney Int..

[54] Visentin S., Grisan E., Zanardo V., Bertin M., Veronese E., Cavallin F., Ambrosini G., Trevisanuto D., Cosmi E. (2013). Developmental programming of cardiovascular risk in intrauterine growth-restricted twin fetuses according to aortic intima thickness. J. Ultrasound Med. Off. J. Am. Inst. Ultrasound Med..

[55] Checkley W., West K.P., Wise R.A., Baldwin M.R., Wu L., LeClerq S.C., Christian P., Katz J., Tielsch J.M., Khatry S. (2010). Maternal vitamin A supplementation and lung function in offspring. N. Engl. J. Med..

[56] Kirkwood B.R., Hurt L., Amenga-Etego S., Tawiah C., Zandoh C., Danso S., Hurt C., Edmond K., Hill Z., Ten Asbroek G. (2010). Effect of vitamin A supplementation in women of reproductive age on maternal survival in Ghana (ObaapaVitA): A cluster-randomised, placebo-controlled trial. Lancet.

[57] Edmond K., Hurt L., Fenty J., Amenga-Etego S., Zandoh C., Hurt C., Danso S., Tawiah C., Hill Z., ten Asbroek A.H.A. (2012). Effect of vitamin A supplementation in women of reproductive age on cause-specific early and late infant mortality in rural Ghana: ObaapaVitA double-blind, cluster-randomised, placebo-controlled trial. BMJ Open.

[58] Berry R.J., Li Z., Erickson J.D., Li S., Moore C.A., Wang H., Mulinare J., Zhao P., Wong L.Y., Gindler J. (1999). Prevention of neural-tube defects with folic acid in China. China-U.S. Collaborative Project for Neural Tube Defect Prevention. N. Engl. J. Med..

[59] Molloy A.M., Brody L.C., Mills J.L., Scott J.M., Kirke P.N. (2009). The search for genetic polymorphisms in the homocysteine/folate pathway that contribute to the etiology of human neural tube defects. Birth Defects Res. Part. A Clin. Mol. Teratol..

[60] Passerini L., Casey G.J., Biggs B.A., Cong D.T., Phu L.B., Phuc T.Q., Carone M., Montresor A. (2012). Increased birth weight associated with regular pre-pregnancy deworming and weekly iron-folic acid supplementation for Vietnamese women. PLoS Negl. Trop Dis..

[61] Leung B.M., Wiens K.P., Kaplan B.J. (2011). Does prenatal micronutrient supplementation improve children’s mental development? A systematic review. BMC Pregnancy Childbirth.

[62] Hales C.N., Barker D.J., Clark P.M., Cox L.J., Fall C., Osmond C., Winter P.D. (1991). Fetal and infant growth and impaired glucose tolerance at age. BMJ.

[63] Barker D.J., Hales C.N., Fall C.H., Osmond C., Phipps K., Clark P.M. (1993). Type 2 (non-insulin-dependent) diabetes mellitus, hypertension and hyperlipidaemia (syndrome X): Relation to reduced fetal growth. Diabetologia.

[64] Yu Y., Arah O.A., Liew Z., Cnattingius S., Olsen J., Sørensen H.T., Qin G. (2019). Maternal diabetes during pregnancy and early onset of cardiovascular disease in offspring: Population based cohort study with 40 years of follow-up. BMJ.

[65] Bieswal F., Ahn M.T., Reusens B., Holvoet P., Raes M., Rees W.D., Remacle C. (2006). The importance of catch-up growth after early malnutrition for the programming of obesity in male rat. Obesity.

[66] Howie G.J., Sloboda D.M., Vickers M.H. (2012). Maternal undernutrition during critical windows of development results in differential and sex-specific effects on postnatal adiposity and related metabolic profiles in adult rat offspring. Br. J. Nutr..

[67] Neel J.V. (1962). Diabetes mellitus: A “thrifty” genotype rendered detrimental by “progress”?. Am. J. Hum. Genet..

[68] Hales C.N., Barker D.J. (1992). Type 2 (non-insulin-dependent) diabetes mellitus: The thrifty phenotype hypothesis. Diabetologia.

[69] Burton M.A., Lillycrop K.A. (2019). Nutritional modulation of the epigenome and its implication for future health. Proc. Nutr. Soc..

[70] Breier B.H., Vickers M.H., Ikenasio B.A., Chan K.Y., Wong W.P. (2001). Fetal programming of appetite and obesity. Mol. Cell Endocrinol..

[71] Gluckman P.D., Hanson M.A. (2004). The developmental origins of the metabolic syndrome. Trends Endocrinol. Metab..

[72] Phipps K., Barker D.J., Hales C.N., Fall C.H., Osmond C., Clark P.M. (1993). Fetal growth and impaired glucose tolerance in men and women. Diabetologia.

[73] Fall C.H., Osmond C., Barker D.J., Clark P.M., Hales C.N., Stirling Y., Meade T.W. (1995). Fetal and infant growth and cardiovascular risk factors in women. BMJ.

[74] Stöger R. (2008). The thrifty epigenotype: An acquired and heritable predisposition for obesity and diabetes?. Bioessays.

[75] Desai M., Babu J., Ross M.G. (2007). Programmed metabolic syndrome: Prenatal undernutrition and postweaning overnutrition. Am. J. Physiol. Regul. Integr. Comp. Physiol..

[76] Bol V.V., Reusens B.M., Remacle C.A. (2008). Postnatal catch-up growth after fetal protein restriction programs proliferation of rat preadipocytes. Obesity.

[77] Agote M., Goya L., Ramos S., Alvarez C., Gavete M.L., Pascual-Leone A.M., Escrivá F. (2001). Glucose uptake and glucose transporter proteins in skeletal muscle from undernourished rats. Am. J. Physiol. Endocrinol. Metab..

[78] Gavete M.L., Martín M.A., Alvarez C., Escrivá F. (2005). Maternal food restriction enhances insulin-induced GLUT-4 translocation and insulin signaling pathway in skeletal muscle from suckling rats. Endocrinology.

[79] Escrivá F., Rodríguez C., Cacho J., Alvarez C., Portha B., Pascual-Leone A.M. (1992). Glucose utilization and insulin action in adult rats submitted to prolonged food restriction. Am. J. Physiol..

[80] Bruss M.D., Khambatta C.F., Ruby M.A., Aggarwal I., Hellerstein M.K. (2010). Calorie restriction increases fatty acid synthesis and whole body fat oxidation rates. Am. J. Physiol. Endocrinol. Metab..

[81] Mackay H., Khazall R., Patterson Z.R., Wellman M., Abizaid A. (2013). Rats perinatally exposed to food restriction and high-fat diet show differences in adipose tissue gene expression under chronic caloric restriction. Adipocyte.

[82] Dulloo A.G., Jacquet J., Seydoux J., Montani J.P. (2006). The thrifty ’catch-up fat’ phenotype: Its impact on insulin sensitivity during growth trajectories to obesity and metabolic syndrome. Int. J. Obes..

[83] Dulloo A.G. (2008). Thrifty energy metabolism in catch-up growth trajectories to insulin and leptin resistance. Best Pract. Res. Clin. Endocrinol. Metab..

[84] Chen L., Magliano D.J., Zimmet P.Z. (2011). The worldwide epidemiology of type 2 diabetes mellitus—Present and future perspectives. Nat. Rev. Endocrinol..

[85] Rughani A., Friedman J.E., Tryggestad J.B. (2020). Type 2 Diabetes in Youth: The Role of Early Life Exposures. Curr. Diab. Rep..

[86] Godfrey K.M., Reynolds R.M., Prescott S.L., Nyirenda M., Jaddoe V.W.V., Eriksson J.G., Broekman B.F.P. (2017). Influence of maternal obesity on the long-term health of offspring. Lancet. Diabetes Endocrinol..

[87] Woo Baidal J.A., Locks L.M., Cheng E.R., Blake-Lamb T.L., Perkins M.E., Taveras E.M. (2016). Risk Factors for Childhood Obesity in the First 1,000 Days: A Systematic Review. Am. J. Prev. Med..

[88] Bianco M.E., Josefson J.L. (2019). Hyperglycemia During Pregnancy and Long-Term Offspring Outcomes. Curr. Diab. Rep..

[89] Smith J., Cianflone K., Biron S., Hould F.S., Lebel S., Marceau S., Lescelleur O., Biertho L., Simard S., Kral J.G. (2009). Effects of maternal surgical weight loss in mothers on intergenerational transmission of obesity. J. Clin. Endocrinol. Metab..

[90] Beyerlein A., von Kries R. (2011). Breastfeeding and body composition in children: Will there ever be conclusive empirical evidence for a protective effect against overweight?. Am. J. Clin. Nutr..

[91] Agostoni C., Baselli L., Mazzoni M.B. (2013). Early nutrition patterns and diseases of adulthood: A plausible link?. Eur. J. Intern. Med..

[92] Reifsnider E., Mendias E. (2012). Early infant feeding influences and weight of children. Childhood Obesity.

[93] Koletzko B., von Kries R., Closa R., Escribano J., Scaglioni S., Giovannini M., Beyer J., Demmelmair H., Anton B., Gruszfeld D. (2009). Can infant feeding choices modulate later obesity risk?. Am. J. Clin. Nutr..

[94] Fall C.H.D. (2011). Evidence for the intra-uterine programming of adiposity in later life. Ann. Hum. Biol..

[95] Heinberg L.J., Thompson J.K. (2009). Obesity in Youth: Causes, Consequences, and Cures.

[96] Langley-Evans S.C., McMullen S. (2010). Developmental origins of adult disease. Med. Princ. Pract..

[97] Sullivan E.L., Grove K.L. (2010). Metabolic imprinting in obesity. Forum Nutr..

[98] Şanlı E., Kabaran S. (2019). Maternal Obesity, Maternal Overnutrition and Fetal Programming: Effects of Epigenetic Mechanisms on the Development of Metabolic Disorders. Curr. Genom..

[99] Hanley B., Dijane J., Fewtrell M., Grynberg A., Hummel S., Junien C., Koletzko B., Lewis S., Renz H., Symonds M. (2010). Metabolic imprinting, programming and epigenetics—A review of present priorities and future opportunities. Br. J. Nutr..

[100] Marshall N.E., Guild C., Cheng Y.W., Caughey A.B., Halloran D.R. (2012). Maternal superobesity and perinatal outcomes. Am. J. Obs. Gynecol..

[101] Jarvie E., Hauguel-de-Mouzon S., Nelson S.M., Sattar N., Catalano P.M., Freeman D.J. (2010). Lipotoxicity in obese pregnancy and its potential role in adverse pregnancy outcome and obesity in the offspring. Clin. Sci..

[102] Friis C.M., Paasche Roland M.C., Godang K., Ueland T., Tanbo T., Bollerslev J., Henriksen T. (2013). Adiposity-related inflammation: Effects of pregnancy. Obesity.

[103] Pacce S., Saure C., Mazza C.S., Garcia S., Tomzig R.G., Lopez A.P., Ribarola L., Krochick G.A. (2016). Impact of maternal nutritional status before and during pregnancy on neonatal body composition: A cross-sectional study. Diabetes Metab. Syndr..

[104] Yu Z., Han S., Zhu J., Sun X., Ji C., Guo X. (2013). Pre-pregnancy body mass index in relation to infant birth weight and offspring overweight/obesity: A systematic review and meta-analysis. PLoS ONE.

[105] Goodwin P.J., Stambolic V. (2015). Impact of the obesity epidemic on cancer. Annu. Rev. Med..

[106] Yang Z., Huffman S.L. (2013). Nutrition in pregnancy and early childhood and associations with obesity in developing countries. Matern. Child. Nutr..

[107] Mohd-Shukri N.A., Duncan A., Denison F.C., Forbes S., Walker B.R., Norman J.E., Reynolds R.M. (2015). Health Behaviours during Pregnancy in Women with Very Severe Obesity. Nutrients.

[108] Picó C., Palou M., Priego T., Sánchez J., Palou A. (2012). Metabolic programming of obesity by energy restriction during the perinatal period: Different outcomes depending on gender and period, type and severity of restriction. Front. Physiol..

[109] Konieczna J., García A.P., Sánchez J., Palou M., Palou A., Picó C. (2013). Oral leptin treatment in suckling rats ameliorates detrimental effects in hypothalamic structure and function caused by maternal caloric restriction during gestation. PLoS ONE.

[110] Delahaye F., Breton C., Risold P.Y., Enache M., Dutriez-Casteloot I., Laborie C., Lesage J., Vieau D. (2008). Maternal perinatal undernutrition drastically reduces postnatal leptin surge and affects the development of arcuate nucleus proopiomelanocortin neurons in neonatal male rat pups. Endocrinology.

[111] Vogt M.C., Paeger L., Hess S., Steculorum S.M., Awazawa M., Hampel B., Neupert S., Nicholls H.T., Mauer J., Hausen A.C. (2014). Neonatal insulin action impairs hypothalamic neurocircuit formation in response to maternal high-fat feeding. Cell.

[112] Ralevski A., Horvath T.L. (2015). Developmental programming of hypothalamic neuroendocrine systems. Front. Neuroendocrinol..

[113] Ramos-Lobo A.M., Teixeira P.D., Furigo I.C., Melo H.M., de M Lyra e Silva N., De Felice F.G., Donato J. (2019). Long-term consequences of the absence of leptin signaling in early life. eLife.

[114] Lillycrop K.A., Burdge G.C. (2011). Epigenetic changes in early life and future risk of obesity. Int. J. Obes..

[115] Bumaschny V.F., Yamashita M., Casas-Cordero R., Otero-Corchón V., de Souza F.S., Rubinstein M., Low M.J. (2012). Obesity-programmed mice are rescued by early genetic intervention. J. Clin. Investig..

[116] Chhabra K.H., Adams J.M., Jones G.L., Yamashita M., Schlapschy M., Skerra A., Rubinstein M., Low M.J. (2016). Reprogramming the body weight set point by a reciprocal interaction of hypothalamic leptin sensitivity and Pomc gene expression reverts extreme obesity. Mol. Metab..

[117] Acosta-Manzano P., Coll-Risco I., Van Poppel M.N.M., Segura-Jiménez V., Femia P., Romero-Gallardo L., Borges-Cosic M., Díaz-Castro J., Moreno-Fernández J., Ochoa-Herrera J.J. (2019). Influence of a Concurrent Exercise Training Intervention during Pregnancy on Maternal and Arterial and Venous Cord Serum Cytokines: The GESTAFIT Project. J. Clin. Med..

[118] Tsakiridis I., Bakaloudi D.R., Oikonomidou A.C., Dagklis T., Chourdakis M. (2020). Exercise during pregnancy: A comparative review of guidelines. J. Perinat. Med..

[119] Mottola M.F. (2009). Exercise prescription for overweight and obese women: Pregnancy and postpartum. Obs. Gynecol. Clin. N. Am..

[120] O’Connor P.J., Poudevigne M.S., Cress M.E., Motl R.W., Clapp J.F. (2011). Safety and efficacy of supervised strength training adopted in pregnancy. J. Phys. Act. Health.

[121] Petrov Fieril K., Fagevik Olsén M., Glantz A., Larsson M. (2014). Experiences of exercise during pregnancy among women who perform regular resistance training: A qualitative study. Phys. Ther..

[122] Roberts J.M., Lain K.Y. (2002). Recent Insights into the pathogenesis of pre-eclampsia. Placenta.

[123] Bonzini M., Coggon D., Palmer K.T. (2007). Risk of prematurity, low birthweight and pre-eclampsia in relation to working hours and physical activities: A systematic review. Occup. Environ. Med..

[124] Price B.B., Amini S.B., Kappeler K. (2012). Exercise in pregnancy: Effect on fitness and obstetric outcomes—A randomized trial. Med. Sci. Sports Exerc..

[125] Soliman A., Lacasse A.A., Lanoix D., Sagrillo-Fagundes L., Boulard V., Vaillancourt C. (2015). Placental melatonin system is present throughout pregnancy and regulates villous trophoblast differentiation. J. Pineal. Res..

[126] Tamanna S., Geraci S.A. (2013). Major sleep disorders among women: (women’s health series). South. Med. J..

[127] Kovac U., Jasper E.A., Smith C.J., Baer R.J., Bedell B., Donovan B.M., Weathers N., Prosenc Zmrzljak U., Jelliffe-Pawlowski L.L., Rozman D. (2019). The Association of Polymorphisms in Circadian Clock and Lipid Metabolism Genes With 2(nd) Trimester Lipid Levels and Preterm Birth. Front. Genet..

[128] Parra O., Sánchez-Armengol Á., Capote F., Bonnin M., Arboix A., Campos-Rodríguez F., Pérez-Ronchel J., Durán-Cantolla J., Martínez-Null C., de la Peña M. (2015). Efficacy of continuous positive airway pressure treatment on 5-year survival in patients with ischaemic stroke and obstructive sleep apnea: A randomized controlled trial. J. Sleep Res..

[129] Pien G.W., Pack A.I., Jackson N., Maislin G., Macones G.A., Schwab R.J. (2014). Risk factors for sleep-disordered breathing in pregnancy. Thorax.

[130] Warland J., Dorrian J., Morrison J.L., O’Brien L.M. (2018). Maternal sleep during pregnancy and poor fetal outcomes: A scoping review of the literature with meta-analysis. Sleep Med. Rev..

[131] Jiang F. (2019). Sleep and Early Brain Development. Ann. Nutr. Metab..

[132] Nakamura Y., Tamura H., Kashida S., Takayama H., Yamagata Y., Karube A., Sugino N., Kato H. (2001). Changes of serum melatonin level and its relationship to feto-placental unit during pregnancy. J. Pineal. Res..

[133] Okatani Y., Okamoto K., Hayashi K., Wakatsuki A., Tamura S., Sagara Y. (1998). Maternal-fetal transfer of melatonin in pregnant women near term. J. Pineal. Res..

[134] Nehme P.A., Amaral F.G., Middleton B., Lowden A., Marqueze E., França-Junior I., Antunes J.L.F., Cipolla-Neto J., Skene D.J., Moreno C.R.C. (2019). Melatonin profiles during the third trimester of pregnancy and health status in the offspring among day and night workers: A case series. Neurobiol. Sleep Circadian Rhythm..

[135] Zhang Y., Sun C.M., Hu X.Q., Zhao Y. (2014). Relationship between melatonin receptor 1B and insulin receptor substrate 1 polymorphisms with gestational diabetes mellitus: A systematic review and meta-analysis. Sci. Rep..

[136] Mendez N., Halabi D., Spichiger C., Salazar E.R., Vergara K., Alonso-Vasquez P., Carmona P., Sarmiento J.M., Richter H.G., Seron-Ferre M. (2016). Gestational Chronodisruption Impairs Circadian Physiology in Rat Male Offspring, Increasing the Risk of Chronic Disease. Endocrinology.

[137] Reiter R.J., Tan D.X., Tamura H., Cruz M.H., Fuentes-Broto L. (2014). Clinical relevance of melatonin in ovarian and placental physiology: A review. Gynecol. Endocrinol..

[138] Zlotos D.P., Jockers R., Cecon E., Rivara S., Witt-Enderby P.A. (2014). MT1 and MT2 melatonin receptors: Ligands, models, oligomers, and therapeutic potential. J. Med. Chem..

[139] Markwald R.R., Melanson E.L., Smith M.R., Higgins J., Perreault L., Eckel R.H., Wright K.P. (2013). Impact of insufficient sleep on total daily energy expenditure, food intake, and weight gain. Proc. Natl. Acad. Sci. USA.

[140] Terrón M.P., Delgado-Adámez J., Pariente J.A., Barriga C., Paredes S.D., Rodríguez A.B. (2013). Melatonin reduces body weight gain and increases nocturnal activity in male Wistar rats. Physiol. Behav..

[141] Onaolapo A.Y., Onaolapo O.J. (2018). Circadian dysrhythmia-linked diabetes mellitus: Examining melatonin’s roles in prophylaxis and management. World J. Diabetes.

[142] Hsu C.-N., Huang L.-T., Tain Y.-L. (2019). Perinatal Use of Melatonin for Offspring Health: Focus on Cardiovascular and Neurological Diseases. Int. J. Mol. Sci..

